# A Dictionary of Practical Medicine; Comprising General Pathology, the Nature and Treatment of Diseases, &c. &c.

**Published:** 1844-04

**Authors:** 


### Art. XIII.

*A Dictionary of Practical Medicine, comprising General Pathology, the Nature and Treatment of Diseases, &c. &c.* By James Copland, m.d. f.r.s., &c. Three Vols. Vols. I, II.—*London*, 1844. 8vo, pp. 1056, 931.

It affords us sincere pleasure to be able to announce to our readers, that the great work, whose title we have just transcribed, has at length arrived at such a stage of its progress, and attained such a form, as to claim a more formal notice from us than we have hitherto given it. Two thirds of the gigantic task undertaken by Dr. Copland are completed, and the first two volumes now stand before us in their own portly size, and fitly apparelled for the bookcase. We are given to unstand that the materials of the remaining volume are in an advanced state of preparation, and that the future parts will appear at shorter intervals than those of the last volume. We trust this may be the case, as the slow and uncertain progress of the work heretofore, has been a cause of great disappointment to the subscribers. It is no satisfactory reply to their discontent, that the magnitude of the labour fully justifies the delay that has taken place, because a positive promise of a much earlier completion was given by the publishers at the time of subscription. If we were to admit the plea of amount of work and difficulty of execution, as justification of slow performance, we could not conscientiously find fault with the author, although his task should not be completed for years; but this amount and this difficulty ought to have been better calculated before the promise was made.

The thing, however, that more immediately concerns us is, that here we have before us two huge volumes out of the three of which the Dictionary is to consist; and when we record this "great fact," we feel it equally a great duty to record our opinion—that, as there is no medical practitioner in this country, old or young, high or low, who will not derive great pleasure and great profit by consulting them, so we think there is no one who should not add them to his library. The information amassed in these volumes is literally enormous, and contemplated simply as an accumulation, it must excite astonishment as the production of an individual; but when it is further considered, that the whole of the materials have been most carefully selected from all existing sources, most patiently studied, valued, winnowed, digested, elaborated, and arranged into compact and simple forms, easily accessible and readily available in practice, it is not easy to point out, in the whole of medical literature, any work by a single hand so much calculated to excite admiration of the industry and talents of the author. In every article contained in the volumes, the reader cannot fail to be struck with the writer's most extensive learning, which has enabled him to collect knowledge from all authorities, ancient and modern, foreign and domestic; and he will, at the same time, be no less surprised than gratified at the singular power which has arranged the whole so lucidly and in such systematic order.

The first volume comprehends the subjects from Abdomen to Furuncle. It contains about 120 articles, and, among others, elaborate treatises on Diseases of the *Blood*, the *Brain*, the *Bronchi*, the *Digestive Canal*; on *Disease in general*, *Dysentery*, *Diseases of the Eye*, and *Fever* in all its forms. The second volume extends from Gall-bladder to Ozæna, and contains about 100 articles. Among these we find treated at great length the subjects of *Hemorrhage*, *Diseases of the Heart*, *Hysterical affections*, *Infection*, *Inflammation*, *Insanity*, Diseases of the *Intestines*, of the *Kidneys*, of the *Larynx and Trachea*, of the *Liver*, of the *Lungs*, &c. Of any single article in the volume, and indeed in the whole work, we think that on Insanity is the largest; it occupies no fewer than 272 columns, or 136 pages, equal to a large octavo volume of ordinary-sized print. We feel bound in justice to add that this article gives so complete a view of the whole subject of insanity in all its aspects, treats so largely of its history, pathology, and management, that it constitutes, in our opinion, the completest treatise on the disease with which we are acquainted.

In comparing the 'Dictionary of Practical Medicine' with its numerous rivals, the work of a large body of contributors, we have always been surprised, and we are more so now than ever, that one man should be able to cope with so many. And looking at the one near us, and which may be regarded as perhaps the best of its class, the *Dictionnaire de Médecine Pratique*, we are naturally reminded of the complimentary couplet addressed to Samuel Johnson on the completion of *his* Dictionary, and which, *mutato nomine*, may, with at least equal propriety, be applied to our author;

And *Copland* well armed like a hero of yore,
Has beat forty Frenchmen and will beat forty more.

---

